# Sugar-Recognizing Ubiquitin Ligases: Action Mechanisms and Physiology

**DOI:** 10.3389/fphys.2019.00104

**Published:** 2019-02-19

**Authors:** Yukiko Yoshida, Tsunehiro Mizushima, Keiji Tanaka

**Affiliations:** ^1^ Ubiquitin Project, Tokyo Metropolitan Institute of Medical Science, Tokyo, Japan; ^2^ Graduate School of Life Science, Picobiology Institute, University of Hyogo, Kobe, Japan; ^3^ Laboratory of Protein Metabolism, Tokyo Metropolitan Institute of Medical Science, Tokyo, Japan

**Keywords:** E3 ubiquitin ligase, ERAD, F-box protein, glycoprotein, N-glycan, SCF complex, sugar chain

## Abstract

F-box proteins, the substrate recognition subunits of SKP1–CUL1–F-box protein (SCF) E3 ubiquitin ligase complexes, play crucial roles in various cellular events mediated by ubiquitination. Several sugar-recognizing F-box proteins exist in both mammalian and plant cells. Although glycoproteins generally reside outside of cells, or in organelles of the secretory pathway, these lectin-type F-box proteins reside in the nucleocytoplasmic compartment. Mammalian sugar-recognizing F-box proteins commonly bind to the innermost position of N-glycans through a unique small hydrophobic pocket in their loops. Two cytosolic F-box proteins, Fbs1 and Fbs2, recognize high-mannose glycans synthesized in the ER, and SCF^Fbs1^ and SCF^Fbs2^ ubiquitinate excess unassembled or misfolded glycoproteins in the ERAD pathway by recognizing the innermost glycans, which serve as signals for aberrant proteins. On the other hand, endomembrane-bound Fbs3 recognizes complex glycans as well as high-mannose glycans, and SCF^Fbs3^ ubiquitinates exposed glycoproteins in damaged lysosomes fated for elimination by selective autophagy. Plants express stress-inducible lectin-type F-box proteins recognizing a wider range of N- and O-glycans, suggesting that the roles of mammalian and plant lectin-type F-box proteins have diverged over the course of evolution to recognize species-specific targets with distinct functions. These sugar-recognizing F-box proteins interpret glycans in the cytosol as markers of unwanted proteins and organelles, and degrade them *via* the proteasome or autophagy.

## Introduction

Ubiquitination occurs in a temporally and spatially specific manner. E3 ubiquitin ligases control ubiquitination by recognizing specific motifs, such as post-translational modifications induced by cell-signaling events or exposed elements that are normally hidden within proteins (Ravid and Hochstrasser, 2008). Cullin-RING E3 ligases (CRLs) are the largest family of E3 enzymes in all eukaryotes (Petroski and Deshaies, 2005). The best characterized CRLs are SCF complexes. Each SCF complex consists of four subunits: a scaffold protein CUL1, a RING protein RBX1, an adaptor protein SKP1, and one of many F-box proteins, which are responsible for substrate recognition. Each F-box proteins consists of an F-box domain, which binds to SKP1, and a divergent carboxy-terminal substrate-binding domain (Zheng et al., 2002). Mammalian F-box proteins have been grouped into three subfamilies according to their substrate-binding domains (Jin et al., 2004): the FBXW and FBXL families possess WD40 repeats and leucine-rich repeats (LRRs) in their binding domains, respectively, whereas the FBXO family does not have any characteristic structural domain(s). The varieties of SCF complexes differ considerably among eukaryotes. For example, there are 22, 72, and 698 F-box proteins in yeast (*Saccharomyces cerevisiae*), human, plant (*Arabidopsis thaliana*), respectively (Hua et al., 2011; Lee et al., 2011; Finley et al., 2012). Furthermore, CUL1, RBX1, and SKP1 are invariable components in the SCF complex in yeasts and metazoans, but the *Arabidopsis* genome encodes 19 SKP1-like proteins (ASK1-19), and some F-box proteins probably interact with several ASK proteins, yielding more diverse SCF complexes in plants (Farras et al., 2001; Gagne et al., 2002; Kuroda et al., 2012).

F-box proteins discriminate among free metabolites and various post-translational modifications in order to correctly ubiquitinate and degrade substrates in cells. For example, the growth-regulating plant hormones auxin/indole-3-acetic acid and jasmonates bind to transport inhibitor response 1 (TIR1) and coronatine-insensitive-1 (COI-1), respectively, to form part of an enlarged protein-binding interface that allows high-affinity interaction with their specific substrate hormone repressors (Tan et al., 2007; Sheard et al., 2010). As a common mechanism in all eukaryotes, many cell-cycle–related F-box proteins recognize phosphorylation in a specific motif in their corresponding substrates (Ang and Wade Harper, 2005; Randle and Laman, 2016). In addition to phosphorylation, other posttranslational modifications are also necessary for ubiquitination of some SCF complex substrates. In mammals, for example, SCF^FBXO22^-KDM4A and SCF^FBXL17^ target methylated p53 and acetylated PRMT1, respectively (Johmura et al., 2016; Lai et al., 2017). In addition, glycosylation is recognized by some F-box proteins in both mammals and plants. In contrast to other posttranslational modifications, the sugar chains of glycoproteins exhibit structural complexity and diversity.

Protein glycosylation occurs in the endoplasmic reticulum (ER) and Golgi, and glycoproteins reside within the lumen of secretory pathway organelles or outside the cell. Because they are separated by an endomembrane or the plasma membrane, sugar chains are normally not accessible to the ubiquitination machinery in the cytosol or nucleus. However, there are several opportunities for glycoproteins to appear in the cytosol. The first possibility is the ER-associated degradation (ERAD) pathway, in which unfolded proteins and orphan subunits are degraded by the proteasome after retrograde transport from the ER to the cytosol (Vembar and Brodsky, 2008). In this case, the N-glycan structures of glycoproteins emerging in the cytosol are high-mannose glycans that are modified by ER-resident enzymes. On the other hand, compounds including silica, monosodium urate, and protein amyloids, which are endocytosed from the extracellular milieu, can injure endosomes and lysosomes, causing glycoproteins modified with complex- or hybrid-type glycans to be leaked from these organelles to the cytosol. Furthermore, some specific-glycans on the surfaces of viruses and bacterial toxins that invaded cells *via* the retro-grade transport pathway may appear in the cytosol. Therefore, sugar chains appearing in the cytosol serve as ubiquitination signal for unwanted proteins and organelles (Yoshida and Tanaka, 2018). In this review, we focus on the substrate recognition mechanisms of sugar-recognizing F-box proteins. We will discuss the differences and similarities in the substrate recognition modes of lectin-type F-box proteins between plants and mammals, from the standpoint of their physiological roles.

## Mechanism of N-Glycan Recognition by Sugar-Recognizing F-Box Proteins

### N-Glycan Recognition by Mammalian Sugar-Recognizing F-Box Proteins

F-box protein recognizing sugar chain 1 (Fbs1), the first ubiquitin ligase component identified as a sugar-recognizing F-box protein, was purified from mouse brain lysate based on its affinity for an N-glycoprotein (Yoshida et al., 2002). Of the 72 human F-box proteins, only three, Fbs1/FBXO2, Fbs2/FBXO6, and Fbs3/FBXO27, have the ability to bind glycoproteins containing high-mannose glycans, which are synthesized in the ER (Yoshida et al., 2003, 2011).

**Figure 1:**
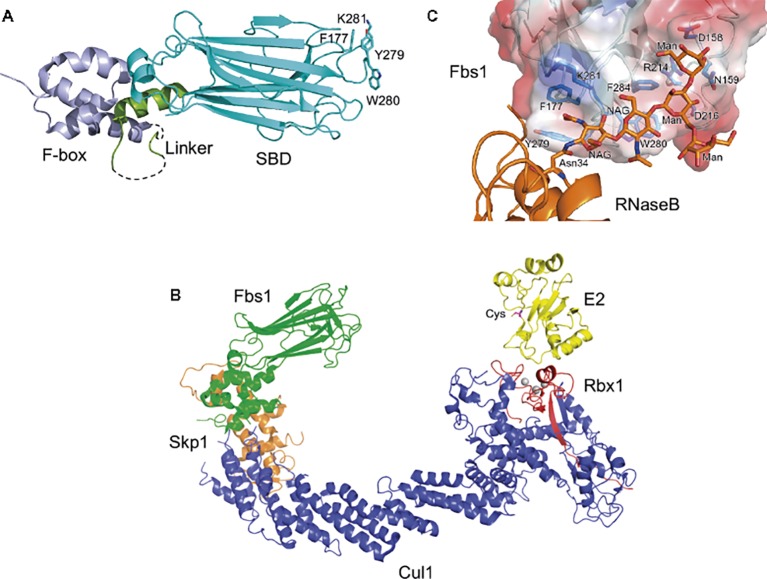
Structure of Fbs1. **(A)** Overall structure of Fbs1. The F-box domain (F-box), linker, and substrate-binding domain (SBD) are shown in violet, green, and cyan, respectively. Dotted lines represent disordered regions. GlcNAc_2_-binding residues are depicted as stick models. **(B)** Model of the SCF^Fbs1^ complex bound to E2. Fbs1, Cul1, Rbx1, Skp1, and E2 are colored green, blue, red, orange, and yellow, respectively. A model of SCF^Fbs1^ was constructed by superposition of the Skp1 subunits from the Skp1–Fbs1, and Skp1–Cul1–Rbx1 structures (Zheng et al., 2002) (PDB ID code 1LDK); the RING-finger domains derived from Rbx1; the c-Cbl subunit of c-Cbl–UbcH7 (Zheng et al., 2000) (PDB ID code 1FBV); and the E2 subunits of c-Cbl-UbcH7. **(C)** Surface representation of the substrate-binding site of the Fbs1 SBD bound to Man_3_GlcNAc_2_ of RNase B. The surface is colored according to the electrostatic potential of the residues (blue, positive; red, negative). Bound RNase B and Man_3_GlcNAc_2_ are orange, and the residues involved in the substrate binding are blue.

These F-box proteins recognize Man_3_GlcNAc_2_ core in N-glycans, but exhibit diverse binding to various glycan structures (Glenn et al., 2008). Structural analysis reveals that the overall architecture of Fbs1 consists of the F-box domain, a linker domain, and a substrate-binding domain (Figure 1A). The substrate-binding domain of Fbs1 is composed of a 10-stranded β-sandwich with an α-helix; it binds Man_3_GlcNAc_2_ through a small hydrophobic pocket in the loops located at the top of the β-sandwich, which protrudes toward E2 (Figure 1B). Man_3_GlcNAc_2_ interacts with Fbs1 through hydrogen bonds and/or hydrophobic interactions (Figure 1C; Mizushima et al., 2004, 2007). The core regions of glycans in native glycoproteins are shielded by the amino acid residues surrounding the glycosylation site, but are exposed upon denaturation. Indeed, Fbs1 and Fbs2 prefer to interact with denatured glycoproteins; thus, exposure of the innermost position of N-glycans upon glycoprotein denaturation serves as a signal of misfolding (Yoshida et al., 2005, 2007; Mallinger et al., 2012). Interestingly, a cytosolic N-Glycanase 1 (NGLY1) also recognizes the same position of glycoproteins and is involved in deglycosylation of various substrates prior to their proteasome-mediated degradation *via* the ERAD pathway (Yamaguchi et al., 2007; Suzuki, 2015). Therefore, ERAD substrates emerging in the cytosol might be denatured.

Although both Fbs1 and Fbs2 preferentially bind high-mannose glycans, Fbs3 binds to glycoproteins modified with complex-type glycans, such as transferrin and LAMP2, as well as high-mannose glycans (Glenn et al., 2008; Yoshida et al., 2017). However, the structural information of Fbs3 is not yet available until now, and the mode of recognition by Fbs3 remains to be elucidated.

### Mammalian Fbs1-Related F-Box Proteins

**Figure 2:**
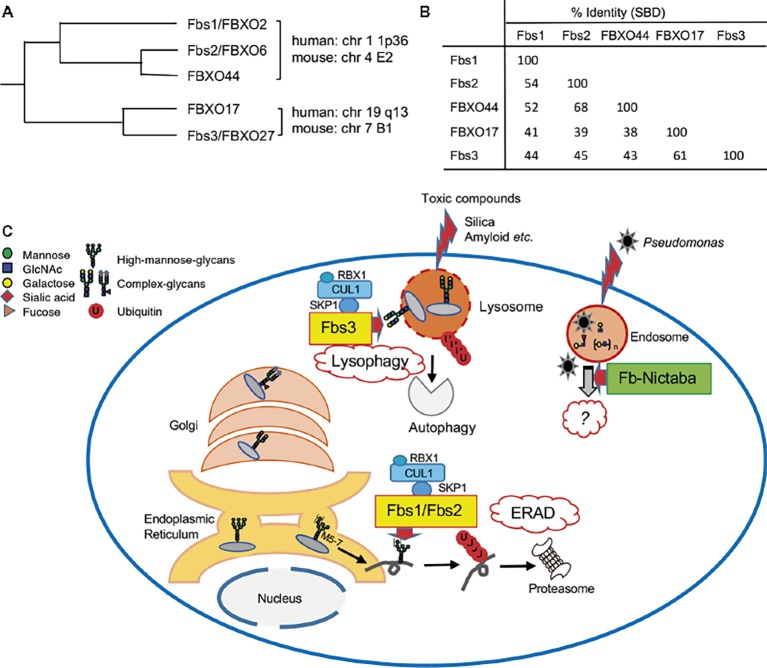
Roles of sugar-recognizing F-box proteins in the cytosol. **(A)** Phylogenetic tree of Fbs1 homologs. Genomic locations of these genes on human or mouse chromosomes are also shown. **(B)** Percentage identities of substrate-binding domain (SBD) of Fbs1 homologs. **(C)** Overview of the functions of sugar-recognizing F-box proteins. Three mammalian F-box proteins (Fbs1, Fbs2, and Fbs3; yellow-colored) form SCF complexes and ubiquitinate glycoproteins in the cytosol, followed by proteasomal or autophagic degradation to maintain cellular homeostasis. They mainly recognize the innermost Man_3_GlcNAc_2_ structure in high-mannose glycans, which are attached in the ER, as a signal of misfolded glycoproteins, and act on excess unassembled subunits or misfolded glycoproteins *via* the ERAD pathway. In contrast to Fbs1 and Fbs2, Fbs3 can bind to complex-type glycans, is targeted to endomembranes via N-myristoylation, and accumulates on organelles such as lysosomes and endosomes ruptured by toxic compounds. SCF^Fbs3^ ubiquitinates exposed the lysosomal glycoprotein LAMP2 to induce autophagy. Dozens of F-box/Nictaba proteins (green) are expressed in plants, but their functions have not been elucidated. These proteins recognize varieties of glycan structure in both N- and O-glycans, and their expression is induced by certain environmental stresses. The *Arabidopsis* F-box/Nictaba protein functions in defense against pathogens such as *Pseudomonas syringae.*

Fbs1/FBXO2 exhibits high sequence similarity with other F-box proteins (Winston et al., 1999; Ilyin et al., 2002). In phylogenetic analysis, these proteins cluster into two groups: one group contains Fbs1/FBXO2, Fbs2/FBXO6, and FBXO44a, and the other contains FBXO17 and Fbs3/FBXO27 (Figure 2A). The genes that encode the proteins each group are arranged in tandem with very short intergenic regions, but the two groups map to different chromosomes in both human and mouse (Ilyin et al., 2002; Yoshida and Tanaka, 2010). These F-box proteins contain a highly homologous F-box domain and a substrate-binding domain (SBD) (Figure 2B), but the long N-terminal region and C-terminal tail are unique to Fbs1 and Fbs2, respectively. The SBD of FBXO44 has 68% identity with the corresponding region of Fbs2; the residues necessary for binding to N-glycans are conserved, and its overall structure is similar to Fbs1 and Fbs2. Nonetheless, FBXO44 has no detectable sugar-binding activity (Glenn et al., 2008; Yoshida et al., 2011). The crystal structure of FBXO44–Skp1 revealed that FBXO44 has different hydrogen bond networks than the four loops from Fbs1 and Fbs2, preventing the formation of the sugar-binding pocket (Kumanomidou et al., 2015; Nishio et al., 2016). Thus far, from a structural perspective, it has been challenging to identify factors that determine the substrate specificity of these isoforms.

### Plant Lectin-Type F-Box Proteins

These Fbs orthologues are encoded in various vertebrate genomes, but other lectin-type F-box proteins are found in plants. High levels of secretory lectins accumulate in plant seeds and vegetative storage tissues, but plants also synthesize small amounts of nucleocytoplasmic lectins in response to specific stress factors and changing environmental conditions. Among nucleocytoplasmic lectins, chimeric proteins that contain F-box domain with Jacalin-related lectins or Nictaba-like lectins were found (Lannoo and Van Damme, 2010). Nictaba, an inducible lectin found in *Nicotiana tabacum* leaves treated with jasmonates (Chen et al., 2002), recognizes Man_3_GlcNAc_2_ core as well as human Fbs family members (Lannoo et al., 2006). However, the three-dimensional conformations and sugar-recognition modes of Nictaba and mammalian Fbs1 differ considerably (Mizushima et al., 2004; Schouppe et al., 2010). *Arabidopsis* and crops such as soybean express dozens of F-box/Nictaba proteins (Delporte et al., 2015; Van Holle et al., 2017), but to date only an *Arabidopsis* F-box/Nictaba protein, At2g02360, has been characterized. The expression of At2g02360 is up-regulated after treatment with salicylic acid, heat stress, or infection with *Pseudomonas syringae.* Plants overexpressing At2g02360 exhibit milder disease symptoms after infection of pathogens, but the molecular mechanisms involved in acquisition of pathogen resistance remain to be elucidated (Stefanowicz et al., 2016). Although At2g02360 shares the highest sequence similarity (64%) to tobacco Nictaba among Arabidopsis F-box/Nictaba proteins, it binds to N- and O-glycans with Galβ1-3GlcNAc and Galβ1-4GlcNAc and poly-N-acetyllactosamine, but not to Man_3_GlcNAc_2_ core (Stefanowicz et al., 2012). It is possible that other F-box/Nictaba proteins have different sugar-binding specificities, but the roles of mammalian and plant lectin-type F-box proteins seem to have diverged substantially over the course of to recognize species-specific targets with distinct functions (Figure 2C).

## Physiological Roles of Mammalian Sugar-Recognizing F-Box Proteins

As described above, Fbs1 and Fbs2 specifically recognize high-mannose glycans, and in particular the innermost structure. Therefore, SCF^Fbs1^ and SCF^Fbs2^ can ubiquitinate excess unassembled or misfolded glycoproteins in the ERAD pathway by recognizing the innermost glycans as signals for aberrant proteins (Figure 2B). On the other hand, Fbs3, which can also interact with glycoproteins modified with complex-type glycans present in organelles downstream of the Golgi or on the cell surface, contributes to maintenance of organelle homeostasis (Figure 2C).

SCF^Fbs1^ and SCF^Fbs2^ are the E3 enzymes responsible for degradation of integrin β1 that is expressed in excess over integrin α chains, or ERAD substrates such as TCRα, asialoglycoprotein receptor H2a, and CFTRΔ508 (Yoshida et al., 2002, 2003; Groisman et al., 2011; Chen et al., 2016; Ramachandran et al., 2016). The ERAD pathway is a ubiquitous protein quality control system in all eukaryotes, and ERAD substrates are generally ubiquitinated by ER membrane-embedded E3s, such as Hrd1 and gp78 (Vembar and Brodsky, 2008). However, Fbs proteins are encoded only in vertebrate genomes. In particular, Fbs1 expression is restricted to a subset of tissues, and therefore its role in in quality control may be tissue-specific.

### Roles of Fbs1 in the Brain and Inner Ear

Originally named neural F-box protein 42 kDa (NFB42) and organ of Corti protein 1 (OCP1), Fbs1 is expressed at high levels in the brain and rodent inner ear (Erhardt et al., 1998; Henzl et al., 2001). Therefore, Fbs1 may function in quality control specifically in the nervous system and inner ear, rather than in the general ERAD system.

NMDA receptors play crucial roles in neuronal development and information storage in the brain, and SCF^Fbs1^ controls the abundance and localization of their specific subunits, GluN1 and GluN2A (Kato et al., 2005; Atkin et al., 2015). Furthermore, Fbs1 attenuates amyloid-β (Αβ) production through ubiquitination of β-secretase (BACE1) and amyloid precursor protein (APP), and the expression of Fbs1 decreases in the brains of Alzheimer’s disease (AD) patients and Tg2576 mice, a well-characterized model of AD (Gong et al., 2010; Atkin et al., 2014). In primary neurons derived from Tg2576 mice, overexpression of Fbs1 promotes the degradation of BACE1, which is essential for Αβ generation, thereby decreasing the Aβ level (Gong et al., 2010). In addition, the total amount of amyloid precursor protein (APP) in the brain of Fbs1-KO mouse is increased but decreased on the surface of cells in hippocampal neurons, indicating that Fbs1 regulates APP protein levels and processing (Atkin et al., 2014). Interestingly, PGC-1α, a transcriptional coactivator involved in control of energy metabolism, regulates Fbs1 expression, and nicotinamide riboside upregulates BACE1 degradation through enhancing PGC-1α expression (Gong et al., 2010, 2013). Compounds such as nicotinamide that stimulate Fbs1 expression/activity may represent candidate therapeutic agents for AD.

Furthermore, Fbs1-knockout mice exhibit age-related hearing loss with cochlear degeneration and high cochlear levels of the inner-ear gap-junction protein GJB2 (Nelson et al., 2007), which is a multi-pass membrane protein that lacks glycans but nonetheless interacts with Fbs1 (Henzl et al., 2004). Fbs1 is an unusually abundant inner ear protein, and exists as a heterodimer with Skp1 but not as a component of the SCF complex, suggesting that its function in inner-ear homeostasis is distinct from that of the conventional SCF complex (Atkin et al., 2015).

Although the expression of Fbs1 is restricted to specific organs under normal condition, recent studies show that expression of Fbs1, like plant Nictaba, is up-regulated in response to some stressors. In the livers of mice with high-fat diet-induced obesity, Fbs1 expression is induced by the IKKβ/NF-κΒ pathway, and SCF^Fbs1^ disrupts glucose homeostasis *via* degradation of the insulin receptor (Liu et al., 2017). Infection with Epstein–Barr virus (EBV) also stimulates Fbs1 expression, and induced SCF^Fbs1^ ubiquitinates and degrades EBV glycoprotein B, thereby decreasing the infectivity of progeny viruses (Zhang et al., 2018). Thus, SCF^Fbs1^ may function in the unusual ERAD system that is induced under certain stresses. The expression of Fbs3, like that of Fbs1, is restricted to specific organs, including the brain. Its levels are very low, but it may be induced by as-yet-undetermined stimuli.

### Roles of Fbs3

Fbs3 has a unique endomembrane localization due to N-myristoylation. This endomembrane localization, together with its glycoprotein-binding activity, is essential for effective recruitment to damaged organelles. Fbs3 accumulates within ruptured lysosomes or endosomes by treated with the lysosomal damage reagent L-leucyl-l-leucine methyl ester (LLOMe), crystalline silica, or latex beads coated with transfection reagents, whereas neither Fbs1 or Fbs2 behaves in this manner (Yoshida et al., 2017). Lysosomes are specialized organelle that contain a variety of digestive enzymes and play a crucial role in autophagy, but damaged lysosomes are themselves eliminated by a special form of autophagy known as lysophagy. Like mitophagy, a well-characterized form of selective autophagy, ubiquitination is prerequisite for lysophagy, but the ubiquitination substrates and molecular mechanisms underlying lysophagy induction have not been elucidated (Maejima et al., 2013). Following lysosomal damage, Fbs3 quickly moves to ruptured lysosomes by detecting exposed glycoproteins, and SCF^Fbs3^ ubiquitinates several lysosomal glycoproteins. Among the lysosomal glycoproteins, ubiquitination of LAMP2 is especially important for recruitment of components of the autophagic machinery, such as p62 and LC3 (Yoshida et al., 2017). Although SCF^Fbs3^ recognizes and ubiquitinates exposed glycoproteins that are normally sequestered in lysosomes following lysosomal damage, resulting in induction of lysophagy, other glycan-recognition systems are also involved in the autophagy-mediated response to damaged endomembranes.

Galectins are β-galactoside–binding lectins, and mostly reside in the cytosol and nucleus. Galectin-8 accumulates on damaged bacteria-containing vesicles and binds directly to NDP52, an autophagy receptor, thereby triggering a specific form of autophagy called xenophagy and restricting the growth of *Salmonella* in cells (Thurston et al., 2012; Li et al., 2013). Galectin-3 also accumulates on damaged organelles, such as phagosomes ruptured by infecting *Mycobacterium* and damaged lysosomes, and interacts with TRIM16, a RING-type ubiquitin ligase. TRIM16 further interacts with the key autophagy regulator ULK1, Beclin1, and ATG16L1, and induces autophagy following their ubiquitination (Chauhan et al., 2016). Thus, several cytosolic glycan-monitoring systems collaborate with the proteasome and autophagy to maintain cellular homeostasis.

## Conclusions and Perspectives

Here we have summarized our current knowledge of the sugar-recognition modes and physiological roles of lectin-type F-box proteins. These proteins recognize cytosolic sugar chains, which are normally present in organelles or extracellularly, as aberrant or harmful signals that trigger ubiquitination, leading to alleviation of deleterious cellular states and maintenance of homeostasis. Mammalian sugar-recognizing F-box proteins commonly bind to the innermost position of N-glycans, and cytosolic NGLY1 removes this sugar degron. Mutations in NGLY1 cause an inherited disorder of the ERAD pathway (Enns et al., 2014), and NGLY1 knockout mice are embryonic lethal (Fujihira et al., 2017). Why would F-box proteins fail to rescue the lethality of NGLY1 deficiency, which is caused by excessive glycoprotein in the cytosol? As with Fbs3, the functions of Fbs1 and Fbs2 may be distinct from the ERAD pathway. For instance, Fbs3 accumulates in ruptured lysosomes and preferentially ubiquitinates LAMP2, which plays a crucial role for lysophagy. Thus, identification of the intrinsic substrates for Fbs1 and Fbs2 is essential for understanding their physiological relevance in maintaining cellular homeostasis. Research of these glycoprotein-related F-box proteins knockout mice in models of disease, including AD, would be also useful for assessing their physiological and pathophysiological roles.

In comparison with mammals, plants have more sugar-recognizing F-box proteins with diverse substrate specificities, but their functions have not been elucidated. The ability of these proteins to form SCF complexes remain to be determined. Among 22 yeast F-box proteins, some function in complexes that lack CUL1, suggesting that not all F-box proteins in plants must form SCF complexes. Future studies should seek to determine their sugar-binding specificities and endogenous interacting proteins, substrates, and components of the complex. The elucidation of the molecular mechanisms underlying induction of sugar-recognizing F-box proteins and promotion of SCF complex formation by various stimuli, as well as detailed analyses of their substrate recognition modes in both plants and mammals, will be crucial to understanding the functions of F-box proteins and cytosolic sugar chains.

## Data Availability

The datasets generated for this study are available on request to the corresponding author.

## Author Contributions

YY and TM wrote the manuscript with supervision from KT. All authors critically appraised the final version of the paper.

### Conflict of Interest Statement

The authors declare that the research was conducted in the absence of any commercial or financial relationships that could be construed as a potential conflict of interest.
